# 应重视经支气管超声引导针吸活检的临床应用

**DOI:** 10.3779/j.issn.1009-3419.2010.05.01

**Published:** 2010-05-20

**Authors:** 殿胜 钟, 清华 周

**Affiliations:** 1 300052 天津，天津医科大学总医院呼吸科 Department of Respiratory Medicine, Tianjin Medical University General Hospital, Tianjin 300052, China; 2 300052 天津，天津医科大学总医院，天津市肺癌研究所，天津市肺癌转移与肿瘤微环境实验室 Tianjin Key Laboratory of Lung Cancer Metastasis and Tumor Microenvironment, Tianjin Lung Cancer Institute, Tianjin Medical University General Hospital, Tianjin 300052, China

常规支气管镜检查技术包括活检和毛刷，主要用于气管、支气管腔内病变的检查。经支气管针吸活检（transbronchial needle aspiration, TBNA）将支气管镜检查的适应症从腔内扩展到腔外和纵隔^[1, 2]^，丰富了支气管镜的检查内容。

20世纪50年代，阿根廷医生Schieppati使用硬质气管镜首次对隆突下淋巴结进行了穿刺；80年代美国王国本（Ko-pen Wang）医生经过多年的研究，将纵隔和肺门淋巴结分成11组，即Wang氏TBNA淋巴结分组，基本解决了常见的纵隔和肺门淋巴结在气管-支气管内穿刺的定位问题；此外，王教授还发明了多种类型的TBNA细胞和组织穿刺针，为TBNA技术的发展、完善和普及做出了重大贡献^[3]^。现在，临床上称之为常规TBNA（conventional TBNA）。随着支气管内超声（endobronchial ultrasound, EBUS）技术的出现、发展，尤其是2000年以后，凸面探头（convex probe, CP）超声支气管镜（CP-EBUS）的研发成功，使实时经支气管超声引导针吸活检（EBUS-TBNA）成为现实。与常规TBNA相比，因为有超声的实时监测，可以提高穿刺的准确性，避免穿刺损伤大的血管，增加了操作者的安全感^[4]^。CP-EBUS支气管镜的出现，使TBNA再次成为了热点，引起了医生们的关注，近几年来，关于EBUS-TBNA临床应用的报道和述评越来越多。

目前，TBNA技术主要用于纵隔及肺门肿大淋巴结或肿物的穿刺活检，在纵隔及肺门淋巴结肿大的病因鉴别诊断方面具有重要和独特的价值。在临床上，TBNA最常用于对非小细胞肺癌进行临床分期以及纵隔肿大淋巴结的鉴别诊断。此外，在其它肺部疾病的诊断方面，如结节病的诊断，也发挥了重要作用^[1, 5-13]^。EBUS-TBNA在诊断肺癌患者纵隔和肺门淋巴结转移的敏感性方面优于常规TBNA、CT和PET，可达95%，特异性为100%，准确性达96%以上^[14]^。

常规TBNA具有操作相对简便、创伤小、费用低等优点^[2]^，需要操作者熟悉纵隔、肺门的解剖结构，根据胸部CT，在气管、支气管内正确选择穿刺位点，有“盲穿”的性质，对于初学者有一定的难度，需要经过反复的操作和积累经验，这样才能提高穿刺的阳性率。

纵隔镜是诊断纵隔淋巴结转移及病变的“金标准”^[15]^，在肺癌临床分期的检查中占有重要的地位。在国外一些较大的胸部疾病诊疗中心，纵隔镜已经成为肺癌患者术前的常规检查。但该检查创伤大，需要在手术室全身麻醉情况下进行。但EBUS-TBNA属于微创技术，可在门诊局部麻醉情况下操作，并发症和创伤远小于纵隔镜检查，并且可以对纵隔镜不能到达的肺门淋巴结进行活检。研究^[6]^显示，EBUS-TBNA对肺癌诊断的敏感性与纵隔镜类似，并可使部分患者免除纵隔镜的检查，从而减少患者的痛苦并降低医疗费用。

EBUS-TBNA可以检测第2、4、7、10-12组淋巴结，内镜超声引导针吸活检（endoscopic ultrasonography guided fineneedle aspiration, EUS-FNA）可探查第5、6、8和9组淋巴结，故EBUS-TBNA联合EUS-FNA几乎可对所有纵隔内淋巴结进行活检^[2]^，这需要呼吸科和消化科医师合作。

EBUS-TBNA具有很高的安全性。一些不良反应与支气管镜操作本身有关，如咳嗽、焦虑等；而EBUS-TBNA本身的不良事件轻微，主要为穿刺部位的出血^[16]^，纵隔内出血和纵隔内气肿极少发生，未见有死亡的报道。

我国于20世纪90年代开展了TBNA技术，并于1999年举办了第一次“TBNA临床应用讲习班”，但真正能坚持开展并获得较好效果的呼吸科医生较少，TBNA技术长期处于一种未被有效使用的状态。近年来，随着CP-EBUS进入中国市场，加上国内陆续举办了一些相关的学术会议和学习班，使更多的呼吸科医生开始关注、了解这项技术，一些大型的医院购置了CP-EBUS，从而使得TBNA技术逐渐升温，关于这个领域的经验和报道也越来越多。

需要指出的是，CP-EBUS价格和维修费用昂贵，普及有一定的困难。此外，外径较粗（6.9 mm），需经口插入，其本身不能取代常规支气管镜的检查，厂家应研发便于临床使用的新一代超声气管镜。再有，目前与EBUS配套使用的穿刺针仅一种，且操作比较麻烦，希望能有更多种类、操作简便的穿刺针问世。

目前，TBNA技术在我国各地发展极不平衡，为了提高EBUS-TBNA的水平，应建立呼吸内镜培训基地，对从业人员进行系统培训，严格考核，实施准入。再有，临床医生应加强与病理科合作，提高检查的阳性率。

为在国内推广EBUS在肺癌诊疗中的应用，我们组织了“ EBUS与肺癌”专题，有幸约请到部分国内在EBUS肺癌应用上有丰富临床经验的专家拨冗撰稿；同时为使本刊读者更多了解该技术进展，我们也约请到了数篇国际稿件以飨读者，其英文内容已刊发于天津市肺癌研究所与著名出版商Wiley-Blackwell合作出版的国际刊物*Thoracic Cancer*（ISSN 1759-7706, www.thoraciccancer.net），中文译文将陆续刊发于本刊^[1, 5-8, 17]^，其中也包括王国本（Ko-pen Wang）教授的文章“TBNA with or without endobronchial ultrasound”^[3]^中文版将后续刊发于本刊。

我们希望通过组织此次专题，达到“抛砖引玉”的效果，推动EBUS在肺癌领域的应用，有更多的临床医师开展、研究此项技术，使广大患者获益。同时本刊欢迎国内同行将EBUS在肺癌中应用的成果撰文给本刊赐稿，与同道交流，共同进步。
